# CD19^+^ CD24^hi^ CD38^hi^ Regulatory B Cells and Memory B Cells in Periodontitis: Association with Pro-Inflammatory and Anti-Inflammatory Cytokines

**DOI:** 10.3390/vaccines8020340

**Published:** 2020-06-26

**Authors:** Helal F. Hetta, Ibrahim M. Mwafey, Gaber El-Saber Batiha, Suliman Y. Alomar, Nahed A. Mohamed, Maggie A. Ibrahim, Abeer Elkady, Ahmed Kh. Meshaal, Hani Alrefai, Dina M. Khodeer, Asmaa M. Zahran

**Affiliations:** 1 Department of Internal Medicine, University of Cincinnati College of Medicine, Cincinnati, OH 45267-0595, USA; 2 Department of Medical Microbiology and Immunology, Faculty of Medicine, Assiut University, Assiut 71515, Egypt; maggie.abdallah@hotmail.com; 3 Department of Oral Medicine and Periodontology Diagnosis and Oral Radiology, Al-Azhar University, Assiut Branch, Assiut 71524, Egypt; Ibrahim.mwafey2020@gmail.com; 4 Department of Pharmacology and Therapeutics, Faculty of Veterinary Medicines, Damanhour University, Damanhour 22511, Egypt; gaberbatiha@gmail.com; 5 Doping Research Chair, Department of Zoology, College of Science, King Saud University, Riyadh 11495, Saudi Arabia; Syalomar@ksu.edu.sa; 6 Department of Medical Biochemistry, Faculty of Medicine, Assiut University, Assiut 71515, Egypt; nahed_eltamawy@yahoo.com; 7 Department of Clinical and Chemical Pathology, Faculty of Medicine, South Valley University, Qena 83523, Egypt; abeer.elkady@gmail.com; 8 Department of Botany and Microbiology, Faculty of Science, Al-Azhar University, Assiut 71524, Egypt; ahmad_meshaal1650@yahoo.com; 9 Department of Medical Biochemistry and Molecular Biology, Faculty of Medicine, Mansoura University, Mansoura 35516, Egypt; hanielrefai@gmail.com; 10 Department of Pharmacology and Toxicology, Faculty of Pharmacy, Suez Canal University, Ismailia 41522, Egypt; dina_khoudaer@pharm.suez.edu.eg; 11 Department of Clinical Pathology, South Egypt Cancer Institute, Assiut University, Assiut 71524, Egypt; asmaam.zahran@yahoo.com

**Keywords:** Breg, periodontitis, inflammatory, cytokines

## Abstract

Regulatory B cells (Bregs) are unique subpopulations of B cells with immune-regulating or immune-suppressing properties and play a role in peripheral tolerance. Due to the current limitations of human Breg studies among periodontal diseases, in the present study, we tried to analyze the change in circulating Bregs, pro-inflammatory, and anti-inflammatory cytokines in patients with periodontitis. Peripheral blood from 55 patients with stage 2 periodontitis and 20 healthy controls was analyzed using flow cytometry to evaluate the frequency of CD19^+^CD24^+^CD38^+^ Breg cells. ELISA was used to assess the serum levels of the pro-inflammatory cytokines, including interleukins (IL)-1β, IL-6, TNF-α, and anti-inflammatory cytokines including IL-10, IL-35, and TGF-β. Increased proportions of Breg cells were observed in patients with stage 2 periodontitis compared to controls. Serum levels of cytokines were significantly higher in patients with periodontitis compared to controls. A significant positive correlation was observed between the frequencies of Breg cells and IL35 levels, IL10 levels, and TGF-β. In conclusion, our results suggest that the increase in peripheral Breg cells and serum cytokine levels among periodontitis patients seems to be closely associated with disease progression, a possible link between periodontitis, and systemic inflammatory process.

## 1. Introduction

Periodontitis is one of the most prevalent chronic immuno-inflammatory diseases. Periodontitis is a common and complex disease of the oral cavity, and up to 60% of the population is affected by chronic periodontitis [1]. The term chronic periodontitis now has changed to periodontitis according to the most recent classification of periodontal diseases. The report introduced by the American Academy of Periodontology added other parameters to the 1999 Classification of Periodontal Diseases and Conditions, such as radiographic bone loss in association with clinical attachment loss [2]. 

The recent advance has dramatically increased our understanding of immune regulatory cells in the pathogenesis of inflammation. The role of immune regulatory cells has gained substantial acknowledgment in light of recent advances in immunology. Studies have shed considerable light on their properties and role in the pathogenesis of inflammation. The active immune cells in human periodontitis contain numerous assortments of infiltrating cells which are arranged abnormally, with an area rich in T lymphocytes and monocytes/macrophages just tangent to the pocket or sulcular epithelium; and a region in the central lamina propria, positioned farther from the microbial agent, which is rich in B cells and plasma cells and lacking in T lymphocytes [3].

Scientists verify the periodontal disease as dense inflammatory infiltrates in the connective tissue. When the resolution is not achieved, the activation of T and B cells are activated to control the chronic inflammation via cytokine release and modulation of osteoclastogenesis [4].

It is currently well accepted that Breg numbers and functional activity often are inversely correlated with the severity and progression of autoimmune disease [5]. However, it has been previously proposed that the differentiation and activation of Bregs occur under the same set of signals that initiate inflammatory responses [6]. The specific surface markers present in human Breg cells remain unclear. Different B cell subsets in circulation have suppressive regulatory functions that are dependent on IL-10, namely CD19^+^ CD24^high^ CD38^high^ Breg cells, CD19^+^ CD24^high^ CD27^+^ Breg cells, and CD25^high^ CD27^high^ CD86^high^ CD1^high^ Breg cells. 

To date, the role of the Breg cells in periodontal homeostasis or disease development has not been thoroughly studied. This study aimed to evaluate the frequency of peripheral Breg cells and related serum cytokine levels among patients with chronic periodontitis.

## 2. Materials and Methods 

### 2.1. Ethics Statement

The study was conducted in accordance with the ethical guidelines of the 1975 Helsinki Declaration and was approved by the local ethics committee (IRB No. AUAREC20190608-10) of the Faculty of Dental Medicine, Al-Azhar University, Assiut Branch. All participants were adults, and all of them provided written informed consent before the collection of samples.

### 2.2. Patient Grouping and Selection Criteria

Seventy-five participants were selected from those attending the out-patient clinic, Oral Medicine and Periodontology Department, Faculty of Dental Medicine, Al-Azhar University, Assiut Branch during the year 2019. Patients were divided into two groups based on their periodontal health status.

The first group, 55 patients with stage two periodontitis exhibiting (peri-implantitis definition) PPD ≥ 6 mm, (clinical attachment loss) CAL ≥ 5mm, and radiographic evidence of bone loss in at least six teeth [7].

The second group, 20 healthy persons exhibiting no signs of periodontal disease, determined by the absence of clinical attachment loss, absence of bleeding on probing or less than 10%, and probing depth less than 3 mm.

Exclusion criteria for patients’ selection

- Patients with any systemic diseases according to the criteria of Modified Cornell Medical Index [8].
- Patients were receiving antibiotics or non-steroidal anti-inflammatory for at least three months before sample collection.
- Patients subjected to previous periodontal therapy during at least six months.
- Patients suffering from any systemic or local inflammatory disease other than gingivitis or periodontitis.
- Smokers.
- Pregnant and lactating women.

### 2.3. Sample Collection

Peripheral blood was collected from control and patient groups in EDTA-coated vacutainer tubes (K2 EDTA) 5.4 mg (BD vacutainer) under standard aseptic conditions. The samples were immediately transferred to the lab for processing and flow cytometric analysis.

### 2.4. Serum Cytokine Measurements

The pro-inflammatory cytokines, including IL-1β, IL-6, TNF-α, and anti-inflammatory cytokines including IL-10 and TGF-β, were measured using ELISA kit (Raybiotech, Norcross, GA, USA) according to the manufacturer instructions. IL-35 serum concentration was measured for all participants using ELISA kit (Glory Science CO., Ltd, Del Rio, TX, USA, Cat No #:99569) according to the manufacturer’s protocol.

### 2.5. Flow Cytometric Detection of Regulatory B and Memory B Cells 

**Figure 1:**
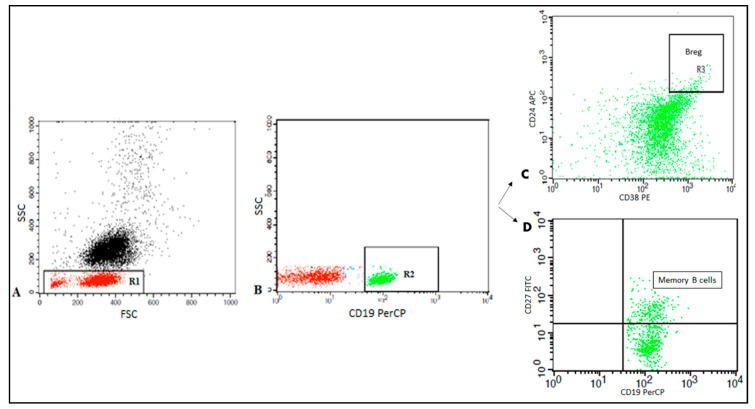
Flow cytometric detection of regulatory B cells and memory B cells. **A**: Forward and side scatter plot was used to define the lymphocytes population (R1). **B**: The CD19^+^ cells (R2) were assessed within the lymphocyte population and then gated. **C**: The expression of CD24 and CD38 was assessed on CD19^+^ cells to define CD19^+^CD24^+high^CD38^+high^ cells (regulatory B cells). **D**: The expression of CD27 was assessed on CD19 to detect memory B cells (CD19^+^ CD27^+^).

Peripheral blood Bregs and memory B cells were enumerated using fluoroisothiocyanate (FITC)-conjugated CD27, phycoerythrin (PE) conjugated CD38, peridinium-chlorophyll-protein (Per-CP)-conjugated CD19, and allophycocyanin (APC)- conjugated CD24. All monoclonal antibodies were from Becton Dickinson (BD) Biosciences, San Jose, CA, USA. After incubation for 15 minutes, red blood cells (RBCs) lysis and washing with phosphate buffer saline (PBS), the cells were resuspended in PBS and analyzed by FACSCalibur flow cytometry with CellQuest software (BD Biosciences). An isotype-matched negative control was used for each sample. Forward and side scatter histogram was used to define the lymphocytes population. CD19^+^ B cells were then gated. Then the expression of CD27, CD38, and CD24 on the CD19^+^ B cells was assessed to detect memory B cells (CD19^+^ CD27^+^) and Bregs (CD19^+^ CD24^high^ CD38^high^) as shown in Figure 1.

### 2.6. Statistical Analysis 

Statistical analyses were performed with GraphPad Prism 8 version 8.0.1 software (Graph Pad Software Inc., San Diego, CA, USA). Qualitative data were expressed as frequency and percentage, while quantitative data were expressed by mean± standard error (SEM). Mann-Whitney analysis was used to detect the statistically significant differences between groups. Spearman’s correlation was used to correlate the studied parameters. A *p*-value ˂ 0.05 was considered significant. 

## 3. Results

### 3.1. Demographic Data

The demographic details of the included groups are summarized in Table 1. 

### 3.2. Frequency of CD19^+^ B Cells, Memory B Cells, and Breg Cells among Periodontitis Patients 

Table 2 comparatively showed the frequency of circulating B cell subsets and Breg cells in patients and healthy control groups. The frequency of CD19^+^ B cells, memory B cells, and Breg cells was significantly higher in the periodontitis patients than in the healthy controls (*p* = 0.04, *p* = 0.0001, and *p* = 0.007), respectively. 

### 3.3. Pro-Inflammatory and Anti-Inflammatory Cytokine Levels in the Studied Groups

The serum levels of cytokines in patients and healthy control groups were further measured, and results were analyzed in Table 2. Significantly higher levels of serum of cytokines IL-6, TNF-α and IL-1β, IL10, IL35, and TGF-β1 were measured in patients with chronic periodontitis when compared to the healthy control group (Table 3). 

### 3.4. Correlation between the Frequency of Breg and Serum Cytokines Levels among Periodontitis Patients

**Figure 2:**
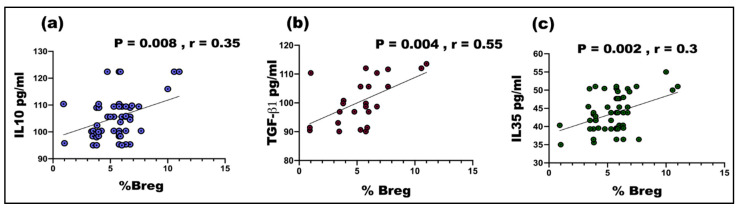
A significant positive correlation between the frequency of Breg cells and (**a**) IL10 levels (r = 0.35, *p* = 0.008), (**b**) TGF-β (r = 0.55, *p* = 0.004), (**c**) IL35 levels (r = 0.3, *p* = 0.002).

As shown in Figure 2, the percentage of CD19^+^CD24^+^CD38^+^ Bregs was positively and significantly correlated with serum levels of IL10 (*p* = 0.008, r = 0.35), TGF- (*p* = 0.004, r = 0.55), and IL35 (*p* = 0.002, r = 0.3). No correlations were found between the Breg frequency and inflammatory cytokines (data not shown). 

## 4. Discussion

Periodontal disease has inflammation as the basis with many confounding factors and hence is referred to as a multifactorial disease. It is a result of host-microbial interaction where the inflammatory component leads to major destruction [1,4]. Recent studies suggest that Foxp3+ Treg cells may have an essential role in regulating periodontal inflammation and alveolar bone resorption in inflamed gingival tissues. Although Bregs have been implicated as a major player in other inflammatory and chronic immune pathologies, very little is known about their involvement in dental inflammatory conditions [9]. In this study, we want to shed some light on Breg’s role in periodontal inflammation and delineate how much their cytokine production contributes to the severity and chronicity of the disease.

Progressive chronic inflammatory periodontal disease in humans shows an increase in the influx of activated B cells that outnumber any other inflammatory cell [10]. Zouali et al. showed that there is a significant increase in local activated lymphocytes in patients presented with periodontitis. He demonstrated that the lesion areas also have significantly increased numbers of plasma cells in comparison to the free areas. [11]. Oliver-Bell et al. [12] used a mouse model to show that B cell-deficient mice were better protected from *P. gingivallis* infection with its subsequent alveolar bone damage. B cells in wild-type mice showed an up-regulation of members of the NF-kB family, pointing to the connection of this pathway in the pathogenesis of periodontitis in this model. Abe et al. [13] used a mechanical model to induce periodontitis in mice. Their data confirmed that B cells are a central player in the development of periodontitis in this model as well, when they reported a significantly milder course of disease in B cell-deficient mice than wild-type controls.

Additionally, we observed that the frequency of memory B cells was significantly decreased among periodontitis patients. In the clinically healthy sulcus, memory B cell trafficking may occur together with the polymorphonuclear cell emigration [14,15]. Such gingival memory B cells are obviously functional, as they can secrete IgG and IgA after in vitro polyclonal stimulation [16]. B cell subsets in gingivitis and periodontitis were initially described by Mahanonda et al., who published that patients of periodontitis show a characteristic distribution of different B cell populations. Identifying plasma cells by being CD138 (+)HLA-DR(low) B cells, he demonstrated that they are the main component of the inflammatory cells mainly at the base of the periodontal pocket and distributed throughout the gingiva, especially toward the advancing edge of the active lesion. Memory B cells identified by being CD27(-) CD38(-) were more prevalent in the interface between the lesion and the rest of the healthy connective tissue, implying that they involve a more protective role of periodontal homeostasis [17].

In the present study, CD19^+^, CD24^+^, and CD38^+^ were used as markers to characterize Breg cells from peripheral blood of periodontitis patients. We observed a significant increase in the peripheral Breg cells in the periodontitis group when compared to healthy controls. Due to the current limitations of human Breg studies in periodontal diseases, evidence supporting the role of Breg in chronic periodontitis needs to be extrapolated entirely from murine studies. 

Bregs have been described as one of the dampening factors of the immune response in the course of inflammation. B10 cells are described as Bregs that negatively regulate the inflammatory responses through the production of IL-10 [18]. The frequency of B10 cells were reported to be significantly higher in periodontal disease lesions when compared to healthy tissues [19]. There is current evidence that the local induction of IL-10 competency of B10 cells could ameliorate both inflammation and bone loss in ligature-induced experimental periodontitis [20]. Furthermore, adoptively transferring Breg cells has been proposed as a potential innovative management technique for periodontal diseases. Research has shown that when used in mice, it leads to a faster resolution of inflammation and less damage to the bone [21]. When a research group extracted B cells and conditioned them ex vivo using a mixture of anti-Tim1, CD40L, and IL-21, then transferred them to mice models of periodontitis, they observed an improvement of bone injury and resorption. [20].

The frequency of Breg cells was positively correlated to serum levels of IL10, IL35, and TGFβ1. Through the production of IL-10, IL-35, and transforming growth factor b (TGF-b), Breg cells suppress immunopathology by prohibiting the expansion of pathogenic T cells and other pro-inflammatory lymphocytes [22]. 

Notably, fewer than 20% of the B cells within these different B cell subsets produce IL-10 and suppress immune responses. Upon in vitro CD40 engagement, CD19^+^, CD24^+^, and CD38^+^ B cells produce high amounts of IL-10 to suppress Th1 differentiation. In addition to inhibiting Th1 responses and Th17 differentiation, CD19^+^, CD24^+^, and CD38^+^ Bregs could also convert CD4+ T cells into Tregs and Tr1 cells [23,24]. 

Although cytokines play a crucial physiological role, they also can induce pathology if secreted inappropriately. In periodontitis, the balance between pro- and anti-inflammation is directed towards pro-inflammatory activity [25]. Accordingly, in our study, we evaluated the change in serum levels of pro-inflammatory and anti-inflammatory cytokines among periodontitis patients, and we found that they had higher significant levels of IL6, IL1b, and TNF compared to healthy individuals. IL-1, IL-6, and TNF-α are pro-inflammatory cytokines that appear to have a crucial role in periodontal tissue destruction [26]. 

IL-1β is an essential mediator of the inflammatory response and is involved in cell proliferation, differentiation, and apoptosis, as well as in promoting alveolar bone destruction of periodontitis [27]. There is evidence that susceptibility to periodontal disease is influenced by genetic polymorphism of the IL-1 gene. Some studies report on an association between IL-1 and the severity of periodontal disease genotype [28]. In a meta-analysis, it is demonstrated that IL-1α and IL-1β genetic variation are significant contributors to chronic periodontitis [25]. 

TGF-β1 is an immunosuppressive cytokine, and up-regulation of TGF-β1 in inflamed gingiva may counterbalance destructive gingival inflammatory responses that are simultaneously taking place in patients with chronic marginal periodontitis. Several previous studies reported the association between the increase in TGF-β1 and chronic periodontitis [26,29,30]. It was suggested that TGF-β1 could be considered as a disease predictive biomarker. Studies indicated that the expression level of TGF-β1 mRNA in the regulatory T cells present in the gingival tissue is correlated with periodontitis [25].

Additionally, it was reported that the levels of IL-6, TNFα, and IL-1β are significantly elevated in the GCF and gingival tissue of periodontitis patients, and the elevated serum levels of IL-6 TNFα and IL-1β decreased after nonsurgical periodontal therapy, resulting in a significant clinical improvement of the periodontal status [29,30].

Interleukin 35 (IL-35) is also secreted by B cells and induces the conversion of human B cells into Breg cells to inhibit antimicrobial immunity through production of IL-35 [31,32]. IL35 may also play a vital role in the immune suppression function of Breg cells. Moreover, a recent study has reported that IL-35 contributes to the dysfunction/exhaustion of T cells and limited antitumor immune responses in Non-Small Cell Lung Cancer [33].

## 5. Future Recommendations

The future research about Breg in periodontitis should include data on the proportion of peripheral and local Th1 and Treg cells. Additionally, it is recommended to correlate the presence of regulatory B cells not only systemically but also locally.

## 6. Conclusions

The increase in peripheral Breg cells and serum cytokine levels among periodontitis patients seems to be closely associated with disease progression, a possible link between periodontitis, and systemic inflammatory process. Circulating Breg cell numbers and serum anti-inflammatory cytokine levels may be used as a reliable additional resource in the determination of periodontitis in the absence of systemic inflammatory disorders. A longitudinal study of serum-cytokine and B reg cell activity in periodontitis patients should be explored as a test for disease progression/resolution.

## Figures and Tables

**Table 1:** Demographic data and clinical parameters of the studied groups.

Parameters	Periodontitis Patients (n = 55)	Healthy Controls (n = 20)
Age (years) mean ± SD	37.46 ± 4.2	35.65 ± 1.6
Gender (male:female)	32:23	13:7
Plaque score (mean) %	2.2	0.58
Bleeding on probing (mean) %	2.5	0.77
Probing depth (PD) mm	5.4	1.4
Clinical attachment loss (CAL) mm	3.3	0

**Table 2:** Frequency of peripheral CD19^+^ B cells, memory B cells, and Breg cells among periodontitis patients and healthy controls.

Percentage (%)	Periodontitis Patients (n = 55)	Healthy Controls (n = 20)	*p*-Value
CD 19^+^B lymphocytes	13.92 ± 4.61	11.27 ± 3.20	0.04
Total memory B cells	21.68 ± 6.06	34.48 ± 5.17	0.0001
Breg cells	5.60 ± 2.59	3.78 ± 0.77	0.007

**Table 3:** Serum levels of cytokine profile between patients and control groups.

Cytokines (pg/mL)	Periodontitis Patients (n = 55)	Healthy Controls (n = 20)	*p*-Value
Pro-inflammatory cytokines			
IL6	125.4 ± 19.03	19.03 ± 4.26	0.0001
TNF-α	202.71 ± 103.8	11.01 ± 7.77	0.0001
IL1β	84.02 ± 11.77	7.03 ± 3.53	0.0001
**Anti-inflammatory cytokines**			
IL10	109.4 ± 7.75	17.90 ± 5.44	0.0001
TGF-β1	99.89 ± 7.80	28.26 ± 6.60	0.0001
IL35	43.06 ± 5.17	23.44 ± 3.39	0.0001
